# The miRNAome of *Opisthorchis viverrini* induced intrahepatic cholangiocarcinoma

**DOI:** 10.1016/j.gdata.2014.08.007

**Published:** 2014-08-28

**Authors:** Jin Peng, Yanjun Feng, Gabriel Rinaldi, Ponlapat Yonglitthipagon, Samantha E. Easley, Therawach Laha, Chawalit Pairojkul, Vajarabhongsa Bhudhisawasdi, Banchob Sripa, Paul J. Brindley, Jason P. Mulvenna, Jeffrey M. Bethony, Jordan L. Plieskatt

**Affiliations:** a Department of Microbiology, Immunology & Tropical Medicine, School of Medicine & Health Sciences, The George Washington University, Washington, DC 20037, USA; b Research Center for the Neglected Diseases of Poverty, School of Medicine & Health Sciences, The George Washington University, Washington, DC 20037, USA; c Infectious Diseases Program, QIMR Berghofer Medical Research Institute, Brisbane, Australia; d Department of Pathology, School of Medicine & Health Sciences, The George Washington University, Washington, DC 20037, USA; e Department of Parasitology, Khon Kaen University School of Medicine, Khon Kaen, 40002, Thailand; f Faculty of Medicine, Khon Kaen University, Khon Kaen, 40002, Thailand; g School of Biomedical Sciences, Faculty of Medicine and Biomedical Sciences, University of Queensland, Brisbane, Australia

**Keywords:** MicroRNA, Cholangiocarcinoma, Intrahepatic cholangiocarcinoma, Opisthorchis viverrini, Microarray

## Abstract

Intrahepatic cholangiocarcinoma (ICC) is an aggressive cancer, arising in the biliary ducts that extend into the liver. The highest incidence of ICC occurs in Southeast Asia, particularly in the Mekong River Basin countries of Thailand, Laos, Cambodia, and Vietnam, where it is strongly associated with chronic infection by the food-borne liver fluke *Opisthorchis viverrini* (OV), one of only three eukaryote pathogens considered Group one carcinogens. Intrahepatic cholangiocarcinoma is usually diagnosed at an advanced stage, with a poor prognosis and survival often less than 24 months. Hence, biomarkers that enable the early detection of ICC would be desirable and have a potentially important impact on the public health in the resource-poor regions where this cancer is most prevalent. As microRNAs (miRNAs) remain well preserved after formalin fixation, there is much interest in developing them as biomarkers that can be investigated using tumor biopsy samples preserved in formalin fixed paraffin embedded (FFPE) tumor blocks. Recently, we reported the first comprehensive profiling of tissue-based miRNA expression using FFPE from the three most common subtypes of OV-induced ICC tumors: moderately differentiated ICC, papillary ICC, and well-differentiated ICC. We observed that each subtype of OV-induced ICC exhibited a distinct miRNA profile, which suggested the involvement of specific sets of miRNAs in the progression of this cancer. In addition, non-tumor tissue adjacent to ICC tumor tissue on the same FFPE block shared a similar miRNA dysregulation profile with the tumor tissue than with normal (non-tumor) liver tissue (individuals without ICC or OV infection). Herein, we provide a detailed description of the microarray analysis procedures used to derive these findings.

## Specifications

**Table:** 

Organism/cell line/tissue	*Homo sapiens*
Sex	11 males 18 females
Sequencer or array type	GPL#18159: Agilent-031181 Unrestricted Human miRNA version 16.0 Microarray
Data format	Raw and Processed
Experimental factors	Three histological grades of Intrahepatic cholangiocarcinoma (ICC) tumor vs. distal non-tumor sections (from the ICC patient) vs. non-ICC “normal” liver biopsy FFPE.
Experimental features	miRNA expression in FFPE samples of three histological grades of ICC tumor tissues when compared to both distal non-tumor samples (from the same ICC patients) and also non-endemic, non-ICC, “normal” liver biopsies.
Consent	Institutional Review Board reviewed
Sample source location	Thailand; Washington D.C., United States

Direct link to deposited data

Deposited data can be found here: http://www.ncbi.nlm.nih.gov/geo/query/acc.cgi?acc=GSE53992.

## Experimental design, materials and methods

### Study samples

Intrahepatic cholangiocarcinoma (ICC) formalin-fixed, paraffin-embedded tissues (FFPE) were obtained from histologically confirmed *O. viverrini* (OV) induced ICC cases archived at the Liver Fluke and Cholangiocarcinoma Research Center, Faculty of Medicine, Khon Kaen University (KKU), Thailand (Table 1). The histological subtypes of ICC cases were determined by Hematoxylin and Eosin (H & E) staining of tissue by pathologists at KKU (BS) and independently confirmed by a pathologist (SEE) at the George Washington University (GWU). The ICC FFPE blocks were then macrodissected into ICC tumor tissue (cholangiocarcinoma tumor tissue or CTT) and distal non-tumor (D-NT) tissue (i.e. tissue distal from dysplasia or frank carcinoma). In addition, 13 non-tumor FFPE blocks (Table 2) derived from liver biopsies of individuals suspected of severe steatosis or steatohepatitis prior to gastric bypass surgery were included as normal non-tumor tissue (N-NT) to assess baseline liver histology of individuals with no ICC and do not reside in an OV endemic region. Details of the specimens, including histological confirmation and preparation of FFPE samples, can be found in [1].

The Institutional Review Boards from both KKU and GWU determined that the samples did not meet the definition of human subjects research, i.e., a living individual about whom an investigator conducting research obtains: a) data through intervention or interaction with the individual or b) private identifiable information. This determination was made since the samples were limited to pre-existing, de-identified specimen analysis labeled with a random code.

### RNA isolation

Total RNA was extracted from each of the FFPE blocks using the miRNeasy FFPE kit (Qiagen) following the manufacturer's protocol [2] and also further detailed in [1]. RNA quality and integrity were examined by spectrophotometry (Nano Drop 2000, Thermo Scientific) and by using an Agilent 2100 Bioanalyzer (RNA 6000 Nano and Small RNA Kits, Agilent). The 260/280 ratios obtained were approximately 2.0, indicating that the RNA was pure. The purified RNA exhibited 260/230 ratios greater than 1.9, or only slightly less than the range of 2.0–2.2 expected, indicating no significant contaminants.

Analysis by the Agilent Bioanalyzer produced RNA integrity numbers (or RIN scores) of two to three, with 28S and 18S peaks largely absent. This value was below the quality criteria (RIN greater than or equal to eight) and indicated degraded RNA. RNA obtained from FFPE tissue samples often has slight modifications and degradation due to the process of formalin fixation and duration of storage is expected [3], [4]. However, due to the stable nature of miRNAs in the FFPE matrix, as shown by us [1], [2] as well as others [5], [6], the extracted RNA was determined to be suitable for subsequent analysis of miRNA profiles by microarray.

### Microarray analysis

Purified RNA from all 46 cases samples including 16 CTT and both types of controls (15, D-NT and 13, N-NT) (Table 1, Table 2) were profiled on the Agilent human miRNA microarray (miRBase Release 16.0). Hybridization and further details are available in the Gene Expression Omnibus (GEO) database and also found in [1].

### Data normalization and analysis

The feature intensity of each sample was transferred to digital data and then log_2_ transformed using Agilent Feature Extraction (V.10.7). All the raw data files in text (.txt) format were analyzed (including statistical analysis) with Agilent GeneSpring software (GX 12.6) (http://genespring-support.com/files/gs_12_6/GeneSpring-manual.pdf) [7].

To determine the presence of aberrantly expressed miRNAs, a new “*project*” and new “*experiment*” were created within GeneSpring (GX 12.6), with *miRNA* selected for analysis type and the data import wizard used for the workflow type. After uploading the raw intensity files into *GeneSpring*, *31181_v18_0 technology* was selected (Table 3). The threshold raw signals were set to 1.0 and 90 percentile and “*shift normalization*” was performed to standardize the statistical parameters across all samples (Table 3). No baseline transformation was performed.

**Fig. 1:**
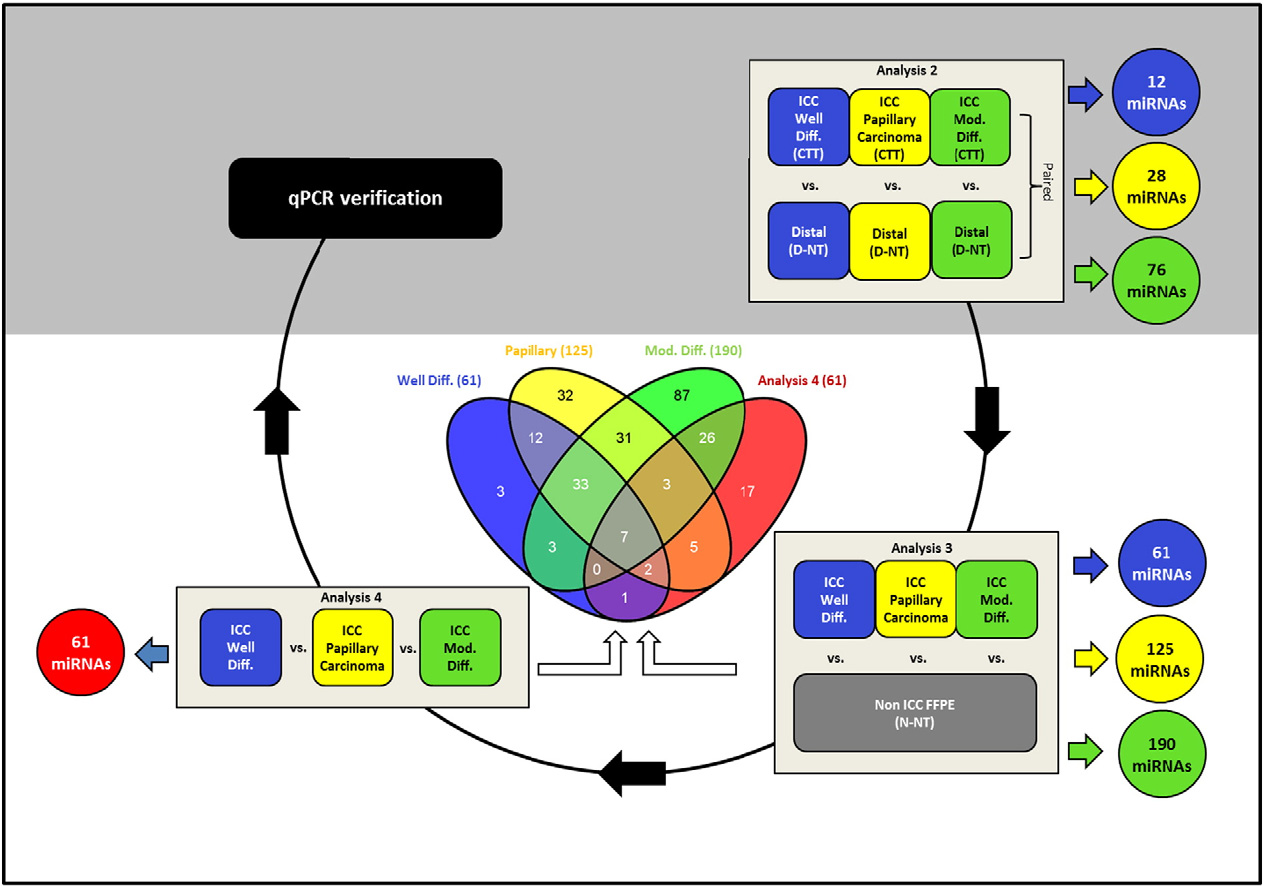
Flow diagram of analyses and associated miRNAs found to be dysregulated. A final qPCR verification step was also completed to confirm the magnitude and expression levels determined by microarray but not described herein [1]. A Venn diagram [8] highlights overlapping miRNAs identified in analysis three and analysis four, yielding seven overlapping miRNAs. Abbreviations are as follows: Intrahepatic cholangiocarcinoma tumor tissue (CTT); distal non-tumor tissue (D-NT); and normal non-tumor tissue (N-NT).

Four different methods were used to analyze this sample set as shown in Fig. 1 and in Table 3. Dysregulated miRNAs were reported as associated with either ICC itself or with ICC stratified by histological subtype as reported in [1].

• *Analysis One*: Normal, non-tumor tissues (N-NT), distal normal tissues (D-NT), and ICC tumor tissues (CCT) samples were analyzed with 3D Principal Components Analysis (PCA), Hierarchical Clustering, and One-way analysis of variance (ANOVA).
• *Analysis Two*: Paired Student's t-test was used to analyze CTT versus D-NT stratified by the histological subtype of ICC.
• *Analysis Three*: Unpaired Student's t-test was used to analyze each histological subtype of ICC tumor (including CTT, D-NT and when available necrotic tissue) versus N-NT, (non-ICC normal, non-tumor tissue).
• *Analysis Four*: One-way ANOVA was used to analyze the differences among the three histological subtypes of ICC FFPE samples (including CTT, necrotic tissue and D-NT).

In all analyses below, subsequent “*interpretations*” were created in GeneSpring (GX 12.6) with the conditions selected, including “*non-averaged*” selected over replicates, and the measurements flagged as “*default*.” In most analyses, unless otherwise noted, the probe sets were filtered by expression value (i.e. 30–336133.0) with at least 50% of the values greater than 30 in any one condition within range.

*Analysis One: CTT versus N-NT versus D-NT by one-way ANOVA*
  1) *In this first experimental design (*Table 3*), the FFPE samples were grouped accordingly: 13 N-NT, 15 D-NT, 2 necrotic samples, and 16 CCT samples (without consideration of histological subtype).*
  2) *For “Quality Control”, the correlation coefficient (CC) and 3D Principal Component Analysis (PCA) scores were used to determine associations among the samples. The CC of sample Y62-N1 (D-NT from a papillary ICC block) was below an acceptable CC (< 0.7) and the sample removed from subsequent analyses. Principle Components Analysis demonstrated that N-NT samples clustered together, and were distinct from the other types of samples (CTT and D-NT). Further details regarding principal component analysis can be found below:*
    • *Algorithm: Principal Components Analysis*
    • *Parameters:*
      ○ *Column indices: [1–45]*
      ○ *Pruning option: [numPrincipalComponents,* *[4]**]*
      ○ *Mean centered: True*
      ○ *Scale: True*
      ○ *3-D scores: True*
      ○ *PCA on: Columns*
  3) *Hierarchical Clustering analysis of the samples' correlations was then conducted on both “entities” and “conditions” on the normalized intensity values after filtering by Euclidean distance metric and Median linkage rule. Hierarchical Clustering details are summarized below:*
    • *Clustering Algorithm: Hierarchical*
    • *Clustered By: Normalized intensity values*
    • *Clustered On: Entities and Conditions*
    • *Similarity Measure: Euclidean*
    • *Linkage Rule: Median*
    • *Cluster Within Conditions: No*
  4) *To analyze the differences between CTT versus D-NT versus N-NT, a one-way ANOVA was conducted (with no post hoc pairwise testing) with asymptotic p-value computation without correction completed. Fold changes (≥ 2.0) were analyzed under pairs of conditions with two minimum numbers of pairs. Fewer differences were observed between tumor tissue (CTT) and distal non-tumor tissue (D-NT) than between D-NT and N-NT, confirming the PCA and Hierarchical Clustering results.*
*Analysis Two: CTT versus D-NT by paired Student's t-test*
  1) CTT samples were again grouped by histological subtype and compared to adjacent non-tumor tissue (D-NT) (see Table 1): i.e., moderately differentiated CTT versus D-NT from the same block, well-differentiated CTT versus D-NT from the same block, and papillary CTT versus D-NT from the same block (Table 3, Box 2 Fig. 1).
  2) To filter probe sets by expression value, in each case the “*entity*” and “*interpretation*” were selected and filtered by raw data values. The lower cut-off value was set to 30 and at least 50% of the values in any one condition within range for well-differentiated CTT versus its D-NT and papillary CTT versus its D-NT. In the case of moderately differentiated CTT versus necrosis versus N-NT, at least 50% of the values in one condition were greater than 20.
  3) In the “*analysis workflow*” in GeneSpring (GX 12.6), paired Student's t-test (*p* < 0.05) was performed to analyze differences among CTT versus D-NT samples of well-differentiated ICC and papillary ICC by asymptotic *p*-value computation without correction for multiple testing. Further a fold change cut-off of 2.0 between conditions (CTT against D-NT) was performed. Only fold changes ≥ 4.0 were analyzed for moderately differentiated CTT versus moderately differentiated necrosis versus moderately differentiated D-NT but statistical significance was not calculated from this limited sample set consisting of only two moderately differentiated CTT samples and a single D-NT sample.
  4) A Venn diagram was plotted to determine the shared dysregulated miRNAs among the groups of analyzed after filtering, statistical analysis, and fold change selection (data not shown).
*Analysis Three: ICC (CTT + D-NT) versus N-NT by unpaired Student's t-test*
  1) All CTT, necrotic, and D-NT samples were grouped (and referred to as “tumor” or “ICC” as a whole) according to histological subtype to generate four different groups of samples: (1) N-NT, (2) well-differentiated ICC, (3) papillary ICC and (4) moderately differentiated ICC (Table 3, Box 3 Fig. 1).
  2) When conducting unpaired Student's t-test (with Benjamini Hochberg False Discovery Rate correction undertaken due to multiple testing), each comparison was given its own *p*-value cut, when testing the three histological subtypes of ICC versus normal liver tissue (N-NT) by asymptotic *p*-value computation: *p* < 0.01 was set for well-differentiated ICC versus N-NT and *p* < 0.005 for papillary and moderately differentiated ICC versus N-NT (*p*-values after Benjamini Hochberg False discovery rate correction). A fold change cut-off of 2.0 between conditions (ICC versus N-NT) was also performed.
  3) A Venn diagram was plotted to show the shared dysregulated miRNAs after filtering, statistical analysis and fold change selection (Center Box, Fig. 1).
*Analysis Four: Histological subtype analysis of ICC by one-way ANOVA*
  1) Here the subtypes of ICC (CTT, necrotic, and D-NT samples were grouped and referred to as “tumor” or “ICC” as a whole) were analyzed against each other (Table 3, Box 4 Fig. 1).
  2) Fold change selection was performed by selecting a cut-off of 2.0 in two pairs of conditions. Further a one-way ANOVA (no pairwise poc hoc testing) with Benjamini Hochberg False Discovery Rate correction was performed among the pairs of conditions by asymptotic *p*-value computation and *p* < 0.05 (*p*-values after Benjamini Hochberg False discovery rate correction).
  3) Significantly dysregulated miRNAs, as determined in Analysis Four, were plotted by Venn diagram and compared to the dysregulated miRNAs determined by Analysis Three for observation of overlap (Center Box, Fig. 1).

## Figures and Tables

**Table 1:** Intrahepatic cholangiocarcinoma (ICC) FFPE cases utilized in the study denoted with associated raw data files and accession numbers. Distal tumor (D-NT) and tumor (CTT) samples types are indicated for each case.

NCBI accession number	Title	Sample type	Sex	Age	Tumor histological grade	Gross Classification	Raw intensity files
GSM1305122	B70-N1	Distal normal	Male	61	Well differentiated	Mass-forming	GSM1305122_US12302349_253118113117_S01_miRNA_1100_Jul11_1_3.txt.gz
GSM1305123	B70-T1	Tumor	GSM1305123_US12302349_253118113117_S01_miRNA_1100_Jul11_1_4.txt.gz
GSM1305124	B79-N1	Distal normal	Male	61	Well differentiated	Periductal infiltrating, invasive intraductal and mixed	GSM1305124_US12302349_253118113117_S01_miRNA_1100_Jul11_2_1.txt.gz
GSM1305125	B79-T1	Tumor	GSM1305125_US12302349_253118113117_S01_miRNA_1100_Jul11_2_2.txt.gz
GSM1305126	B83-N1	Distal normal	Female	53	Well differentiated	Mass-forming	GSM1305126_US12302349_253118113117_S01_miRNA_1100_Jul11_2_3.txt.gz
GSM1305127	B83-T1	Tumor	GSM1305127_US12302349_253118113117_S01_miRNA_1100_Jul11_2_4.txt.gz
GSM1305108	B99-N1	Distal normal	Male	48	Well differentiated	Mass-forming	GSM1305108_US12302349_253118112956_S01_miRNA_1100_Jul11_2_1.txt.gz
GSM1305109	B99-T1	Tumor	GSM1305109_US12302349_253118112956_S01_miRNA_1100_Jul11_2_2.txt.gz
GSM1305110	Y42-N1	Distal normal	Male	61	Well differentiated	Mass-forming	GSM1305110_US12302349_253118112956_S01_miRNA_1100_Jul11_2_3.txt.gz
GSM1305111	Y42-T1	Tumor	GSM1305111_US12302349_253118112956_S01_miRNA_1100_Jul11_2_4.txt.gz
GSM1305106	B90-N1	Distal normal	Male	58	Well differentiated	Mass-forming	GSM1305106_US12302349_253118112956_S01_miRNA_1100_Jul11_1_3.txt.gz
GSM1305107	B90-T1	Tumor	GSM1305107_US12302349_253118112956_S01_miRNA_1100_Jul11_1_4.txt.gz
GSM1305100	B91-Nec1	Necrotic	Male	63	Moderately differentiated	Mass forming	GSM1125485_US12302349_253118112871_S01_miRNA_107_Sep09_2_1.txt.gz
GSM1305101	B91-T1	Tumor	GSM1125486_US12302349_253118112871_S01_miRNA_107_Sep09_2_2.txt.gz
GSM1305102	Y70-N1	Distal normal	Female	63	Moderately differentiated	Mass forming	GSM1125487_US12302349_253118112871_S01_miRNA_107_Sep09_2_3.txt.gz
GSM1305103	Y70-T1	Tumor	GSM1125488_US12302349_253118112871_S01_miRNA_107_Sep09_2_4.txt.gz
GSM1305116	Y56-N1	Distal normal	Female	56	Papillary carcinoma	Periductal infiltrating, invasive intraductal and mixed	GSM1305116_US12302349_253118112957_S01_miRNA_1100_Jul11_2_1.txt.gz
GSM1305117	Y56-T1	Tumor	GSM1305117_US12302349_253118112957_S01_miRNA_1100_Jul11_2_2.txt.gz
GSM1305118	Y62-N1	Distal normal	Male	57	Papillary carcinoma	Periductal infiltrating, invasive intraductal and mixed	GSM1305118_US12302349_253118112957_S01_miRNA_1100_Jul11_2_3.txt.gz
GSM1305119	Y62-T1	Tumor	GSM1305119_US12302349_253118112957_S01_miRNA_1100_Jul11_2_4.txt.gz
GSM1305114	B40-N1	Distal normal	Male	64	Papillary carcinoma	Mass forming	GSM1305114_US12302349_253118112957_S01_miRNA_1100_Jul11_1_3.txt.gz
GSM1305115	B40-T1	Tumor	GSM1305115_US12302349_253118112957_S01_miRNA_1100_Jul11_1_4.txt.gz
GSM1305129	Y83-N1	Distal normal	Female	51	Papillary carcinoma	Mass forming	GSM1305129_US12302349_253118113118_S01_miRNA_1100_Jul11_1_2.txt.gz
GSM1305130	Y83-T1	Tumor	GSM1305130_US12302349_253118113118_S01_miRNA_1100_Jul11_1_3.txt.gz
GSM1305131	Y88-N2	Distal normal	Female	58	Papillary carcinoma	Periductal infiltrating, invasive intraductal and mixed	GSM1305131_US12302349_253118113118_S01_miRNA_1100_Jul11_1_4.txt.gz
GSM1305132	Y88-T1	Tumor	GSM1305132_US12302349_253118113118_S01_miRNA_1100_Jul11_2_1.txt.gz
GSM1305133	Y89-N	Distal normal	Female	60	Papillary carcinoma	Mass forming	GSM1305133_US12302349_253118113118_S01_miRNA_1100_Jul11_2_2.txt.gz
GSM1305134	Y89-Nerc	Necrotic	GSM1305134_US12302349_253118113118_S01_miRNA_1100_Jul11_2_3.txt.gz
GSM1305135	Y89-T	Tumor	GSM1305135_US12302349_253118113118_S01_miRNA_1100_Jul11_2_4.txt.gz
GSM1305138	Y93-N1	Distal normal	Male	63	Papillary carcinoma	Periductal infiltrating, invasive intraductal and mixed	GSM1305138_US12302349_253118113119_S01_miRNA_1100_Jul11_1_3.txt.gz
GSM1305139	Y93-T1	Tumor	GSM1305139_US12302349_253118113119_S01_miRNA_1100_Jul11_1_4.txt.gz
GSM1305140	Y96-N1	Distal normal	Female	64	Papillary carcinoma	Mass forming	GSM1305140_US12302349_253118113119_S01_miRNA_1100_Jul11_2_1.txt.gz
GSM1305141	Y96-T1	Tumor	GSM1305141_US12302349_253118113119_S01_miRNA_1100_Jul11_2_2.txt.gz

**Table 2:** Additional FFPE cases, normal non-tumor tissue (N-NT) from non-ICC gastric bypass patients, utilized in the study denoted with raw data file names and accession numbers.

NCBI accession number	Title	Sample Type	Raw intensity files
GSM1305096	3325A	Non-ICC, N-NT	GSM1305096_US12302349_253118112955_S01_miRNA_1100_Jul11_1_1.txt.gz
GSM1305097	3337A	Non-ICC, N-NT	GSM1305096_US12302349_253118112955_S01_miRNA_1100_Jul11_1_2.txt.gz
GSM1305098	3356A	Non-ICC, N-NT	GSM1305096_US12302349_253118112955_S01_miRNA_1100_Jul11_1_3.txt.gz
GSM1305099	3377A	Non-ICC, N-NT	GSM1305096_US12302349_253118112955_S01_miRNA_1100_Jul11_1_4.txt.gz
GSM1305104	3575A	Non-ICC, N-NT	GSM1305104_US12302349_253118112956_S01_miRNA_1100_Jul11_1_1.txt.gz
GSM1305105	3578A	Non-ICC, N-NT	GSM1305105_US12302349_253118112956_S01_miRNA_1100_Jul11_1_2.txt.gz
GSM1305112	3641A	Non-ICC, N-NT	GSM1305112_US12302349_253118112957_S01_miRNA_1100_Jul11_1_1.txt.gz
GSM1305113	3707A	Non-ICC, N-NT	GSM1305113_US12302349_253118112957_S01_miRNA_1100_Jul11_1_2.txt.gz
GSM1305120	3563A	Non-ICC, N-NT	GSM1305120_US12302349_253118113117_S01_miRNA_1100_Jul11_1_1.txt.gz
GSM1305121	3564A	Non-ICC, N-NT	GSM1305121_US12302349_253118113117_S01_miRNA_1100_Jul11_1_2.txt.gz
GSM1305128	3869A	Non-ICC, N-NT	GSM1305128_US12302349_253118113118_S01_miRNA_1100_Jul11_1_1.txt.gz
GSM1305136	3967A	Non-ICC, N-NT	GSM1305136_US12302349_253118113119_S01_miRNA_1100_Jul11_1_1.txt.gz
GSM1305137	4565A	Non-ICC, N-NT	GSM1305137_US12302349_253118113119_S01_miRNA_1100_Jul11_1_2.txt.gz

**Table 3:** Key parameters utilized in four independent analyses.

1.0 threshold to raw signals, 90 percentile shift normalization to all samples, 31181_v18_0 technology							
Analysis 1	Analysis 2			Analysis 3			Analysis 4
N-NT vs. CTT vs. D-NT vs. necrosis	CTT vs. D-NT			ICC (CTT + D-NT) vs. N-NT			Well differentiated vs. papillary vs. moderately differentiated
	Well differentiated CTT vs. D-NT	Papillary CTT vs. D-NT	Moderately CTT vs. Nec vs. D-NT	Well differentiated (CTT + D-NT) vs. N-NT	Papillary (CTT + D-NT + Nec) vs. N-NT	Moderately differentiated (CTT + D-NT + Nec) vs. N-NT	Well differentiated (CTT + D-NT) vs. Papillary (CTT + D-NT + Nec) vs. Moderately differentiated (CTT + D-NT + Nec)
3D PDA	Raw value > 30		Raw value > 20	Raw value > 30			Raw value > 30
Raw > 30	Paired t-test, *p* < 0.05	Paired t-test, *p* < 0.05	N/A	unpaired t-test, Benjamini Hochberg FDR correction, *p* < 0.01	unpaired t-test, Benjamini Hochberg FDR correction, *p* < 0.005	unpaired t-test, Benjamini Hochberg FDR correction, *p* < 0.005	Fold change (FC) > 2 in 2 pairs
Hierarchical clustering,	FC > 2		FC > 4 in 2 pairs	FC > 2			One-way ANOVA, Benjamini Hochberg FDR correction, p(corr) < 0.05
Euclidean distance metric, Median linkage rule							
One-way ANOVA, *p* < 0.05							

Abbreviations are as follows: Intrahepatic cholangiocarcinoma tumor tissue (CTT); distal non-tumor tissue (D-NT); and normal non-tumor tissue (N-NT).
